# Signaling Properties and Pharmacological Analysis of Two Sulfakinin Receptors from the Red Flour Beetle, *Tribolium castaneum*

**DOI:** 10.1371/journal.pone.0094502

**Published:** 2014-04-09

**Authors:** Sven Zels, Heleen Verlinden, Senne Dillen, Rut Vleugels, Ronald J. Nachman, Jozef Vanden Broeck

**Affiliations:** 1 Molecular Developmental Physiology and Signal Transduction, Department of Biology, KU Leuven, Leuven, Vlaams-Brabant, Belgium; 2 Areawide Pest Management Research Unit, Southern Plains Agricultural Research Center, USDA, College Station, Texas, United States of America; Wake Forest University, United States of America

## Abstract

Sulfakinin is an insect neuropeptide that constitutes an important component of the complex network of hormonal and neural factors that regulate feeding and digestion. The key modulating functions of sulfakinin are mediated by binding and signaling via G-protein coupled receptors. Although a substantial amount of functional data have already been reported on sulfakinins in different insect species, only little information is known regarding the properties of their respective receptors. In this study, we report on the molecular cloning, functional expression and characterization of two sulfakinin receptors in the red flour beetle, *Tribolium castaneum*. Both receptor open reading frames show extensive sequence similarity with annotated sulfakinin receptors from other insects. Comparison of the sulfakinin receptor sequences with homologous vertebrate cholecystokinin receptors reveals crucial conserved regions for ligand binding and receptor activation. Quantitative reverse transcriptase PCR shows that transcripts of both receptors are primarily expressed in the central nervous system of the beetle. Pharmacological characterization using 29 different peptide ligands clarified the essential requirements for efficient activation of these sulfakinin receptors. Analysis of the signaling pathway in multiple cell lines disclosed that the sulfakinin receptors of *T. castaneum* can stimulate both the Ca^2+^ and cyclic AMP second messenger pathways. This in depth characterization of two insect sulfakinin receptors may provide useful leads for the further development of receptor ligands with a potential applicability in pest control and crop protection.

## Introduction

Sulfakinins (SKs) are a family of invertebrate neuropeptides that are involved in the complex regulation of feeding and digestion in insects [1]. SKs are widely distributed throughout insect orders; peptidomic studies have revealed the presence of SK in dipterans [2]–[4], coleopterans [5], orthopterans [6], hymenopterans [7] and hemipterans [8], [9]. Most SKs possess a sulfated tyrosyl residue in their characteristic C-terminal heptapeptide core sequence D/EYGHMRFamide [10], although nonsulfated SKs occur *in vivo* as well [11]. The first insect SKs were isolated from head extracts of the Madeira cockroach *Leucophaea maderae* and showed myotropic activity on the isolated cockroach hindgut [12], [13]. These leucosulfakinins display sequence similarity with the vertebrate neuropeptides cholecystokinin (CCK) and gastrin [12], [13]. SKs are currently classified as both structural and functional arthropod homologs of vertebrate gastrin and CCK [10]. The first SK encoding precursor sequence was characterized in the fruit fly *Drosophila melanogaster*. Three putative neuropeptides are encoded by the *Drome*-SK cDNA. Two peptides, drosulfakinin-I and drosulfakinin-II showed extensive sequence homology to other insects SKs, while a third unrelated peptide encoded by the precursor is currently termed drosulfakinin-0 [14]. The sulfakinin prepropeptide of *T. castaneum* encodes two possible neuropeptides flanked by dibasic cleavage sites, namely the true sulfakinin GEEPFDDYGHMRFamide and the sulfakinin-like peptide QTSDDYGHLRFamide [5]. The discovery and characterization of the first SK peptides and their coding sequences in the late 1980s has triggered an active search for the different physiological functions of SKs in insects.

SK is a potent myotropic neuropeptide and can act on multiple tissues of the insect body. Most studies were conducted on isolated hindguts [12], [13], [15]–[17], but in addition, SK was shown to cause contractions of foregut [17], [18], heart [19] and body wall [20] muscles. In contrast to the plethora of stimulatory effects on visceral muscle contractions, myoinhibitory effects on different parts of the *D. melanogaster* gut were reported for both sulfated and nonsulfated forms of drosulfakinins [21]. SK also inhibited contractions of the heart, ejaculatory duct and oviduct in the giant mealworm beetle, *Zophobas atratus* [22]. SK is a clear inhibitor of food uptake in multiple insect species. Food intake dropped significantly upon injection of SK in the desert locust *Schistocerca gregaria* [23], the German cockroach *Blattella germanica* [17], the blow fly *Phormia regina* [24] and the red flour beetle *Tribolium castaneum* [25]. RNAi knockdown of the sulfakinin precursor or the sulfakinin receptor evoked an increased consumption of food in the Mediterranean field cricket *Gryllus bimaculatus* [26] and in *T. castaneum* [27], [28]. In addition to its role as a satiety regulator, SK also participates in the regulation of digestive enzyme release [29], [30]. Furthermore, SK is also involved in larval and adult locomotion [20], [31], [32], odor preference [31] and synaptic growth [33] in *D. melanogaster*.

Despite the extensive collection of functional research on the peptide itself, little attention has been paid to the SK signaling system(s). Multiple SK receptors have been annotated and all of them are rhodopsin-like G-protein coupled receptors (GPCRs). Up to date, only two SK receptors, both from *D. melanogaster,* have been functionally characterized. The first SK receptor (DSK-R1) was activated by a sulfated drosulfakinin-I analog in a dose-dependent manner [34]. Both drosulfakinin-I and drosulfakinin-II were able to activate a second SK receptor (designated as the CCK-like receptor, CCKLR-17D1) from *D. melanogaster* [20]. A study using a combination of RNAi, overexpression and rescue mutants of *D. melanogaster* showed that synaptic growth promotion by SK, utilizes the CCKLR-17D1 and that this receptor couples to the cAMP pathway via the Gα_s_ subunit of the G-protein [33]. The only other protostomian animal with a characterized CCK-like signaling system is the nematode *Caenorhabditis elegans*. The cloned *C. elegans* CCKlike receptor was activated by two endogenous peptides derived from the neuropeptide-like protein 12. These peptides show structural similarity to vertebrate CCK and insect SK peptides and contain the C-terminal hexapeptide YRPLQFamide in which the tyrosine residue can be sulfated [35]. No further details concerning CCK/SK-like signaling systems in protostomians are known up to date. Therefore, detailed characterization of the SK-activated GPCRs in different insect species is needed to provide useful insights into the mechanisms underlying SK action.

In this study, we analyzed the signaling properties of two sulfakinin receptors from *T. castaneum*. Both sulfated and nonsulfated SKs were tested for their ability to activate these receptors. In addition, we investigated the functional capacity of the SK receptors to regulate Ca^2+^ and/or cyclic AMP (cAMP) second messenger pathways. We also tested multiple SK-like peptide analogs in order to identify which amino acid residues are crucial for receptor activation. Quantitative reverse transcriptase PCR (qRT-PCR) analysis was performed to study the distribution of both receptor-encoding transcripts in different tissues.

## Materials and Methods

### Animal Rearing and Dissections

Beetles were reared under dark conditions at 30°C on Petri dishes of 140 mm diameter containing wheat flour and brewer’s yeast. Adult beetles were sexed based on the presence of a small patch of short bristles on the inside of the first pair of legs in males, according to the *T. castaneum* rearing protocol (http://bru.gmprc.ksu.edu/proj/tribolium/wrangle.asp) [36]. Tissues from sexually mature *T. castaneum* were dissected under a binocular microscope in phosphate buffered saline (PBS) (NaCl 137 mM, KCl 2.7 mM, Na_2_HPO_4_ 10 mM, KH_2_PO_4_ 1.76 mM; pH 7.2) and snap-frozen in liquid nitrogen. Tissues of at least fifteen animals were pooled for all samples. Central brain, optic lobes, gut, salivary glands, fat body and testes were dissected from adult males; ovaries were dissected from adult females. For all paired tissues the entire pair was dissected from each beetle.

### Receptor Transcript Distribution

Dissected tissues were homogenized and RNA was extracted using the RNAqueous Micro Kit (Ambion) according to the manufacturer’s protocol. A DNase treatment to digest remaining genomic DNA was included in the protocol. Total RNA was reverse transcribed to cDNA using SuperScriptIII reverse transcriptase (Invitrogen) as recommended by the kit and diluted ten-fold before use as template in the quantitative (real-time) reverse transcription PCR (qRT-PCR).

Primer pairs were designed using Primer Express software (Applied Biosystems) and subjected to melting curve analysis for verification of specificity and efficiency of amplification (95°C for 15 s, followed by 60°C for 60 s and increase in temperature in 0.7°C increments from 60°C to 95°C). Additionally, amplification products of PCR reactions were analyzed for the presence of one single band by means of gel electrophoresis on a 1% agarose gel. Sequencing of the bands confirmed their identity. All primers used in the qRT-PCR analysis are listed in Table 1.

**Table 1:** Nucleotide sequences of primers used for qRT-PCR analysis of SK receptors.

Name	Forward Primer	Reverse Primer
***Trica-*** **SKR1**	5′-AGGCCTTTCCACAGTTTGGT-3′	5′-GCCATGCTCTTGCTCATTCC-3′
***Trica-*** **SKR2**	5′-AAACGCCGAACGCAGTCT-3′	5′-ACGGCGAAGAGCATTTTTATG-3′
***Trica-*** **RPs3**	5′-ACCTCGATACACCATAGCAAGC-3′	5′-ACCGTCGTATTCGTGAATTGAC-3′
***Trica*** **-Actin**	5′-CGTGTCTTTTCAAACGTAAATACTAATCA-3′	5′-GCACATACCGGATCCATTGTC-3′

For qRT-PCR Fast SYBR Green Master Mix (Applied Biosystems) as per manufacturer’s instruction and the StepOnePlus Real-Time PCR system (Applied Biosystems) were used. Fast SYBR Green Master Mix contains the fluorescent ROX as a passive reference. All samples were measured in duplicate and all plates contained a no template control for all primer pairs to check for possible contamination of the master mix. The following PCR program was used: 95°C for 600 s, followed by 40 cycles of 95°C for 3 s and 60°C for 30 s. The relative quantity of target cDNA was quantified by means of the ΔΔCT method including normalization to a calibrator on all PCR plates and an endogenous control. Prior to the assay, a list of seven housekeeping genes was analyzed using the GeNorm software [37], revealing the most stable expression for ribosomal protein 3 (RPs3) and β-actin with respect to sex and tissue. These transcripts were thus selected for further use as endogenous controls [38].

### Cloning and Sequence Analysis of *T. castaneum* SK Receptors

Both full length receptor sequences were amplified by PCR using *T. castaneum* whole body cDNA and Advantage II polymerase mix (Clontech). The specific oligonucleotide primers used for the *T. castaneum* SK receptor 1 were: *Forward* 5′-CCAATGTCAGAAGTGGAAATGAAC-3′ and *Reverse* 5′- CTAAACACGATCTTCGGCTTCC-3′, while the *T. castaneum* SK receptor 2 was amplified by means of the *Forward* 5′-CCAATGGACTGGGCTGAAAACTC and *Reverse* 5′-TTATCTACAAAAGTCGGCATTTTCCGAG-3′ primers (Sigma-Aldrich). The PCR program used to amplify both receptors consisted of an initial denaturation step of 60 s at 95°C, followed by 30 cycles of [30 s at 95°C, 60 s at 60°C, 180 s at 68°C] and a final elongation step of 300 s at 68°C. PCR fragments were analyzed on a 1% agarose gel and purified using the GenElute Gel Extraction Kit (Sigma-Aldrich). Amplified DNA fragments were subsequently cloned into a pcDNA3.1/V5-His-TOPO TA expression vector (Invitrogen) and transformed into One Shot TOP10 chemically competent *Escherichia coli* cells (Invitrogen). Transformed bacteria were cultivated overnight at 37°C on Luria-Bertani (LB) agar plates (35 g/l, Sigma-Aldrich) containing ampicillin (10 mg/ml, Invitrogen). Single colonies were transferred to 5 ml LB medium (25 g/l, Sigma-Aldrich) with 25 μl ampicillin (10 mg/ml, Invitrogen) and grown overnight at 37°C in a shaking incubator. Plasmid DNA was isolated using the GenElute HP Plasmid Miniprep Kit (Sigma-Aldrich) and inserts were sequenced using an ABI PRISM 3130 Genetic Analyzer (Applied Biosystems) according to the ABI PRISM BigDye Terminator Ready Reaction Cycle Sequencing Kit (Applied Biosystems) protocol. Bacterial cells harboring an expression vector with the correct receptor insert were transferred to 100 ml LB medium (25 g/l, Sigma-Aldrich) with 500 μl ampicillin (10 mg/ml, Invitrogen) and grown overnight at 37°C in a shaking incubator. Plasmid DNA was isolated by means of the EndoFree Plasmid Maxi Kit (Qiagen). Online tools were used to assess the receptor sequences for correct transmembrane topology and putative modification sites. Transmembrane topology was predicted by TMHMM Server v. 2.0 (http://www.cbs.dtu.dk/services/TMHMM/). Putative modification site analysis included N-linked glycosylation site prediction by NetNGlyc 1.0 server (http://www.cbs.dtu.dk/services/NetNGlyc/), palmitoylation site prediction by GSS-PALM version 4.0 (http://csspalm.biocuckoo.org/online.php) and phosphorylation site prediction by GPS version 3.0 (http://gps.biocuckoo.org/online.php).

### Peptides

Peptides were synthesized by means of the FMOC methodology under previously described conditions [12], [24], [39]. Peptide mass was confirmed by MALDI-TOF mass spectrometry and the amount of peptide was quantified by amino acid analysis. All peptides used in this study are listed in Table 2.

**Table 2:** Relative activation of both *T. castaneum SK* receptors by 29 different peptides.

Peptide	Sequence	% *Trica*-SKR1*	% *Trica*-SKR2*
s*Trica*-SK(5–13)	FDDY(SO_3_H)GHMRFa	100.0	100.0
ns*Trica*-SK(5–13)	FDDYGHMRFa	29.9	23.5
s*Locmi*-SK	pQLASDDY(SO_3_H)GHMRFa	75.0	79.9
ns*Locmi*-SK	pQLASDDYGHMRFa	22.2	18.2
2003[φ1]wp-2	FDDYGHMRAa	8.2	15.6
2004[φ1]wp-1	FDDYGHMAFa	5.9	7.3
2005[φ1]wp-3	FDDYGHARFa	5.1	11.1
2006[φ1]wp-1	FDDYGAMRFa	2.3	3.5
2007[φ1]wp-1	FDDYAHMRFa	30.6	25.6
2008[φ1]wp-2	FDDAGHMRFa	10.1	12.1
2009[φ1]wp-1	DDYGHMRFa	27.2	17.1
2010[φ1]wp-1	DYGHMRFa	7.6	8.6
2011[φ1]wp-1	YGHMRFa	26.3	21.2
2053[φ1]wp-4	GHMRFa	13.3	44.6
2052[φ1]wp-3	HMRFa	43.4	33.4
2051[φ2]wp-2	MRFa	5.0	6.5
2076[φ1]wp-2	FDDYGHMRa	25.8	19.7
1569[φ2]wp-4	PVDY(SO_3_H)DRPIMAFa	6.2	13.4
1567[φ1]wp-5	SPVDY(SO_3_H)DRPIMAFa	23.6	33.7
1432-2[φ]wp-6	SPVDYDRPIMAFa	16.4	11.2
1591-1[φ1]wp-3	EAY(SO_3_H)GH[Nle]KFa	71.4	13.7
1598-2[φ2]wp-4	EY(SO_3_H)GH[Nle]KFa	55.6	50.8
1658[φ1]wp-9	DDY(SO_3_H)GH[Nle]RFa	96.2	97.8
1678A[φ1]wp-4	DY(SO_3_H)RPLQFa	40.8	32.6
1678B[φ1]wp-6	DGY(SO_3_H)RPLQFa	28.5	19.9
1679[φ1]wp-4	pQPSY(SO_3_H)DRDIMSFa	16.5	16.6
1835[φ2]wp-4	SDDY(SO_3_H)GHMRFa	58.2	41.4
1011[φ2a]wp-7-4	GGDDQFDDYGHMRFa	40.1	57.4
1070[φ2]wp-2	FDD[Asu]GHMRFa	58.7	42.3

Each peptide was added to a well of a 96-well plate at a final concentration of 1 μM. The response of the sulfated SK from *T. castaneum* [s*Trica-*SK (5–13)] at this concentration was used as 100% value for each separate 96-well plate. Response of all other peptides was expressed relatively to this 100% response level. At least three biological repeats (each performed in duplicate) were used to quantify the relative response of each peptide at each receptor. These screens were performed in CHO-WTA11 cells.

### Cell Culture and Transfections

General pharmacological studies were performed in Chinese Hamster Ovary (CHO) WTA11 cells, stably coexpressing apoaequorin, a zeocin resistance gene and the promiscuous Gα_16_, which couples most agonist-induced GPCRs to the phospholipase C and Ca^2+^ pathway irrespective of their natural signaling cascade. CHO-PAM28 cells stably expressing apoaequorin and a puromycin resistance gene but not Gα_16_ and HEK293T cells (Invitrogen) were used to determine possible effects on Ca^2+^ and/or cAMP second messenger systems, respectively. All cell lines were cultured in Dulbecco’s Modified Eagles Medium nutrient mixture F12-Ham (DMEM/F12; Invitrogen) supplemented with 1% penicillin/streptomycin (10000 units/ml penicillin and 10 mg/ml streptomycin in 0.9% NaCl; Invitrogen) to prevent bacterial contamination of gram-positive and gram-negative bacteria. In CHO-WTA11 culture medium 250 μg/ml zeocin (Invitrogen) was added as a selection marker, while CHO-PAM28 culture medium was supplemented with 5 μg/ml puromycin to select cells stably expressing apoaequorin. All cell culture media were supplemented with 10% fetal bovine serum (inactivated at 65°C; Sigma-Aldrich).

Cells were cultured *in vitro* as a monolayer at 37°C with a constant supply of 5% CO_2_ and were subcultivated twice a week. Transfections of cells were performed in T75 flasks at 60–80% confluency. Transfection reagent for CHO cells was prepared by combining 3.75 ml Opti-MEM I (Invitrogen), 7.5 μg plasmid DNA and 18.75 μl of Plus Reagent (Invitrogen). After gently mixing and 5 min incubation at room temperature 45 μl of Lipofectamine LTX was added to the mixture. This transfection medium was incubated for 30 min at room temperature and added dropwise to the cells, supplemented with 3 ml of fresh culture medium. HEK 293T cells were cotransfected with a receptor construct (6 μg) and CRE-luciferase reporter construct (3 μg), consisting of the open reading frame of the luciferase gene, downstream of a multimerized cAMP-response-element (CRE) and promoter [38]. After transfection, cells were grown overnight before an additional 20 ml of culture medium was supplemented. Cells were again incubated overnight for a final growth phase before luminescence screens were performed.

### Aequorin-luminescence Assay

The aequorin luminescence assay was used to measure Ca^2+^ signaling in CHO cell lines. Cells were detached using phosphate buffered saline (PBS) containing 0.2% EDTA and collected in 10 ml of DMEM/F-12 (without phenol red, with L-glutamine and 10 mM HEPES; Gibco). The viable cells were quantified using a NucleoCounter NC-100 (Chemometic). Cells were pelleted by 4 min centrifugation at 800 rpm at room temperature and resuspended in DMEM/BSA (DMEM/F-12 without phenol red, with L-glutamine and 10 mM HEPES, 0.1% bovine serum albumin) at a concentration of 5×10^6^ cells/ml. Cells were then shielded from light and Coelenterazine H (Invitrogen) was added at a final concentration of 5 μM. Cells were gently shaken at room temperature for 4 h under dark conditions, allowing the aequorin holoenzyme to be reconstituted. After a 10-fold dilution in DMEM/BSA, cells were incubated another 30 min under the same conditions. Pharmacological ligands were dissolved in DMEM/BSA and 50 μl of ligand was added to wells of a 96-well plate. Wells containing 50 μl of DMEM/BSA were used as a negative control, while wells containing 1 μM of ATP served as positive control. Incubated cells were added to the wells of the 96-well plate and light emission was measured for 30 s using a Mithras LB940 (Berthold Technologies). After 30 s Triton X-100 (0.2% in DMEM/BSA) was added, lysing the cells and thus serving as an internal reference. Light emission was measured for an additional 8 s after Triton X-100 was introduced in the wells. Light emission from each well was calculated relative to the total response (ligand+Triton X-100) using the output file of Mikrowin2000 software (Mikrotek). Further analysis was done in Graphpad Prism 5.

### Luciferase Reporter-gene Assay

The luciferase reporter-gene assay was used to quantify positive or negative coupling of the receptor to cAMP in HEK293T cells. Cotransfected HEK293T cells were detached and the viable cells were quantified using the NucleoCounter NC-100 (Chemometic) as described for CHO cells. Cells were pelleted and resuspended at a concentration of 10^6^ cells/ml in DMEM/F-12 containing 200 μM 3-isobutyl-1-methylxanthine (IBMX; Sigma-Aldrich). Fifty μl of this cell suspension was added to the wells of a 96-well plate. Pharmacological ligands were dissolved in either DMEM/F-12 containing 200 μM IBMX to study stimulatory effects or in DMEM/F-12 containing 200 μM IBMX and 20 μM of NKH477 (a water-soluble analog of forskolin; Sigma-Aldrich) for the quantification of inhibitory effects. DMEM/F-12 containing 200 μM IBMX was used as a negative control, while DMEM/F-12 containing 200 μM IBMX and 20 μM of NKH477 was used as a positive control. Fifty μl of ligand was added to wells of the 96-well plate already containing 50 μl of cell suspension. After dispensing of cells and ligands the 96-well plate was incubated for 3–4 h at 37°C and 5% CO_2_. Visualization of luciferase enzymatic activity was quantified by the addition of 100 μl SteadyLite Plus (Perkin Elmer), after which the plate was gently shaken at room temperature for 15 min under dark conditions. Light emission, resulting from the luciferase activity, was measured for 5 s/well using a Mithras LB940 (Berthold Technologies). Data were analyzed as described for CHO cells.

## Results and Discussion

### Cloning and Sequence Analysis of *T. castaneum* SK Receptors

Two separate cDNA fragments coding for the SK receptors were amplified by PCR. The open reading frame of the *Trica*-SK receptor 1 (*Trica*-SKR1) consisted of 1668 nucleotides encoding a 555 amino acid receptor (Figure S1) with a calculated molecular weight of 63.09 kDa. When compared to GenBank sequence KC161573 three additional nucleotides, encoding a glutamic acid (E545) were found in the cloned receptor sequence. Transmembrane topology prediction revealed the presence of seven hydrophobic regions forming the transmembrane segments (TM1-7) characteristic of GPCRs. The open reading frame of the *Trica*-SK receptor 2 (*Trica*-SKR2) is shorter than that of the *Trica*-SKR1 spanning a total of 1263 nucleotides. It encodes a 420 amino acid long receptor (Figure S2) with a calculated molecular weight of 47.91 kDa. Comparison of the cloned open reading frame with GenBank sequence XM967657 revealed three mutations (C756 = > T756, A852 = > C852 and A984 = > T984), all of them silent. The amino acid sequence of the cloned *Trica*-SKR2 thus is identical to the one found in GenBank. Seven putative transmembrane regions were identified by transmembrane topology prediction. A possible disulfide bond can be formed between two cysteine (C125 and C203) residues in extracellular loop (ECL) I and ECL II in the *Trica*-SKR1. In the *Trica*-SKR2, this disulfide bond can be created between C112 in ECL I and C190 in ECL II. Such a disulfide bond is present in both types of vertebrate CCK receptors and helps in stabilizing the extracellular ligand binding pocket [40]. The possibility of a disulfide bridge in both *T. castaneum* SK receptors hints towards a similar stabilizing structure being present in both these receptors.

N-linked glycosylation of N7, N32, and N38 on the extracellular N-terminus is possible in the *Trica*-SKR1, while N6 and N11 represent putative glycosylation sites on the extracellular N-terminus of the *Trica*-SKR2. In the *Trica*-SKR1, palmitoylation of the vicinal cysteines C495 and C496 after TM7 may anchor this C-terminal region to the plasma membrane. No such palmitoylation site is predicted at the C-terminal end of the *Trica*-SKR2. In the *Trica*-SKR1, putative intracellular phosphorylation sites for protein kinase C include S256, S265, S267, S278, S280, S309, S317, S322, S339, S384, S406, S503, T510, S539 and T540 while protein kinase A can use S267, T279, S280 and S539 as putative substrate sites. The intracellular loops (ICLs) and C-terminus of *Trica*-SKR2 contain a lower number of predicted consensus sites for these kinases. Possible protein kinase C sites are S262, S265, S305, S381 and S413 and the single predicted protein kinase A susceptible residue is T294. Putative G protein-coupled receptor kinase (GRK) target sites in the *Trica*-SKR1 are S268, S280, S283, S285, S288, S376, T389, T532 and S544, while the *Trica*-SKR2 may become phosphorylated by GRKs at S283 and S413. In ECLII of the *Trica*-SKR1, a methionine (M196) and arginine (R199) residue may prove to be important for efficient binding of the sulfated tyrosine residue of SK. The methionine residue possibly interacts with the aromatic ring of the tyrosine, while an ionic interaction between the negatively charged sulfate moiety and the positively charged arginine residue might further stabilize the binding [41], [42]. In ECL II of the *Trica*-SKR2, only an arginine (R191) residue is present to aid in binding the negatively charged sulfate moiety, but no methionine is observed.

**Figure 1:**
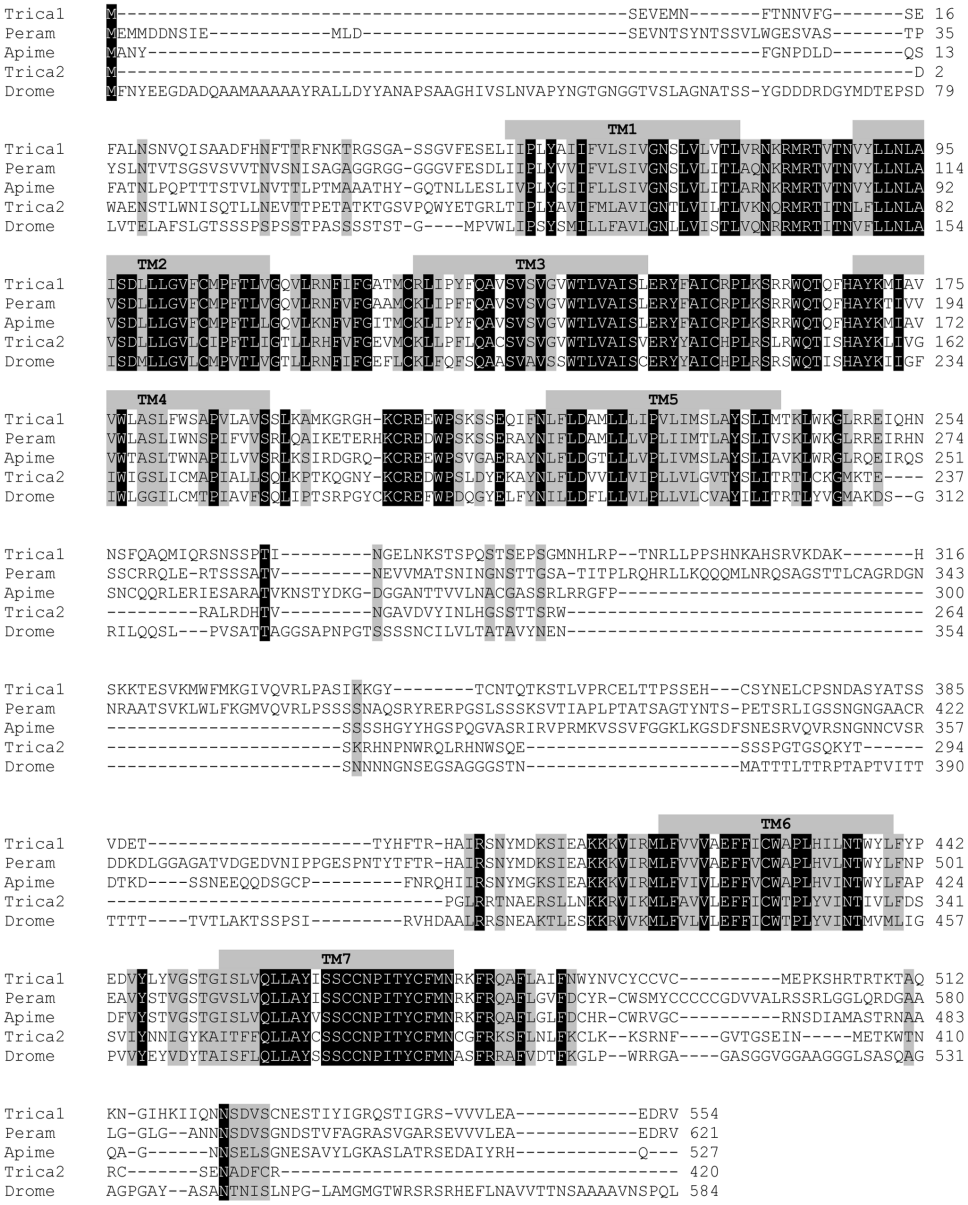
Amino acid sequence alignment of *T. castaneum* SK receptors. Amino acid alignment of *Trica*-SKR1 (Trica1: GenBank Acc no AGK29938) and *Trica*-SKR2 (Trica2: GenBank Acc no XP_972750) against the homologous receptors from *Drosophila melanogaster* (Drome: GenBank Acc no NP_001097023), *Apis mellifera* (Apime: GenBank Acc no XP_003250082) and *Periplaneta americana* (Peram: GenBank Acc no AAX56942). Identical residues between the receptors are shown as white characters against black background. Conserved residues are shaded. Putative transmembrane domains are indicated by gray bars (TM1-7).

tBLASTx (http://blast.ncbi.nlm.nih.gov/blast/) revealed similarities of the cloned receptors with SK or CCKlike receptors from other insects. The *Trica*-SKR1 shows significant resemblance to amongst others *Periplaneta americana* perisulfakinin receptor (54.6% identity; GenBank Acc No AY865608), *Apis mellifera* cholecystokinin like receptor (52.3% identity; GenBank Acc No XM_006562370), *Apis florea* cholecystokinin like receptor (42.9% identity; GenBank Acc No XM_003689506) and *Anopheles gambiae* CCK1 receptor (39.9% identity; GenBank Acc No XM_309215). The *Trica*-SKR2 appears to be most similar to *Nilaparvata lugens* neuropeptide receptor A9 (53.1% identity; GenBank Acc No AB817292), *Pediculus humanus corporis* CCK receptor (47.4% identity; GenBank Acc No XM_004536444) and SK receptors from several *Drosophila* species, including *D. melanogaster* CCKlike receptor (36.7% identity; GenBank Acc No NM_001103553). Both *T. castaneum* SK receptors show a reciprocal identity of 33.6%. Multiple sequence alignment of both *T. castaneum* SK receptors with the *P. americana* perisulfakinin receptor, *A. mellifera* cholecystokinin-like receptor and *D. melanogaster* CCK-like receptor protein sequences revealed that the most conserved regions are TM1-7, ICL I and II and ECL I and II, while substantial variation is present in other regions of the receptors (Figure 1).

### Receptor Transcript Distribution of *T. castaneum* SK Receptors

**Figure 2:**
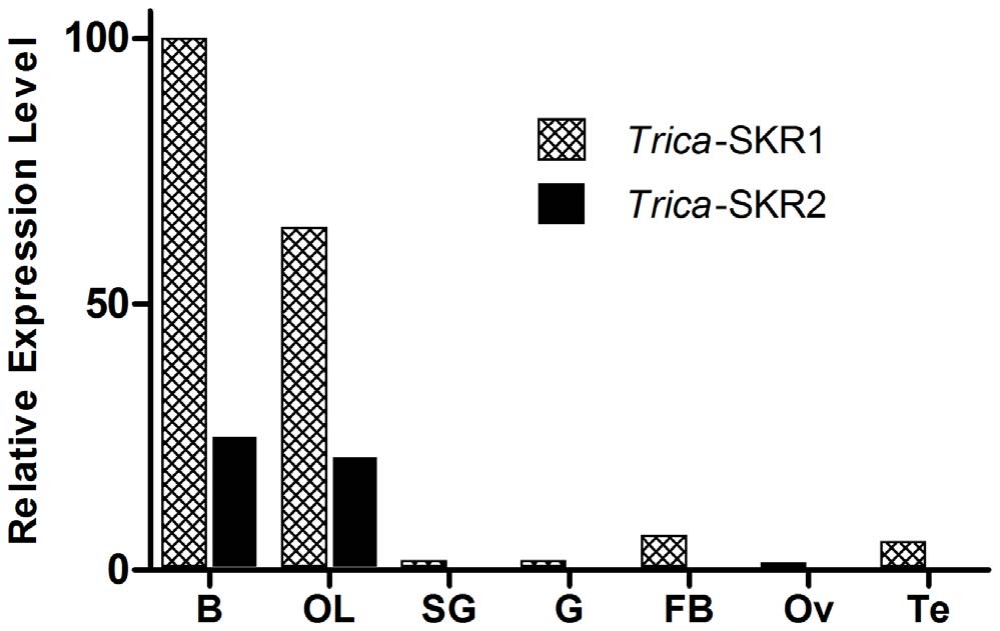
Transcript distribution profile of *Trica-*SKR1 and *Trica*-SKR2. Quantification of transcript levels by qRT-PCR in seven different tissues from adult *T. castaneum*. The data represent samples of central brain (n = 15), optic lobes (n = 15), salivary glands (n = 15), gut (n = 15), fat body (n = 20), testes (n = 50) and ovaries (n = 50), normalized relative to β-actin and ribosomal protein 3 (RPs3) transcript levels. Abbreviations: B = central brain, OL = optic lobes, SG = salivary glands, G = gut, FB = fat body, Te = testes, Ov = ovaria.

A receptor transcript distribution analysis by qRT-PCR reveals that both *T. castaneum* SK receptors show a similar expression pattern (Figure 2). Transcript levels are highest in the central brain, followed by the optic lobes. In all other examined tissues, SK receptor transcript levels appeared much less abundant or did not reach the detection limit. In the cockroach, *P. americana,* expression of the SK receptor in different thoracic and abdominal ganglia was shown by RT-PCR. In addition, immunoblotting and immunocytochemistry showed that the SK receptor was present on gut membrane fractions and in a few peripheral neuronal cell bodies, like the dorsal unpaired median neurons of *P. americana* [43]. These neurons are possibly involved the regulation of an animal’s general activity level [44]. In our study, expression of SK receptors in the gut seemed limited, when compared to the brain and optic lobes. From all other sampled tissues, expression of sulfakinin receptors appeared to be most prominent in the fat body. The presence of a SK receptor transcript in the fat body might hint towards a possible involvement of SK in the energy storing and releasing processes that take place in the fat body. Adipokinetic hormone plays a central role in this energy metabolism, inducing the release of fatty acids and sugars from the fat body as energy source [45]. Since SK is known to induce satiety [17], [23], [24], [26]–[28], it might be possible that it aids in replenishing the energy stores in the fat body and thus counteracts AKH. An antagonistic action of SK and AKH in energy metabolism was suggested earlier in a study in *P. americana* [43].

Expression levels were slightly higher in the central brain than in the optic lobes for both receptors. In different species, sulfakinin immunoreactivity was predominantly found in the central brain [24], [46]–[49] and other nervous tissues [47], including ingluvial ganglia and axons projecting from these ganglia to the anterior midgut and pyloric sphincter [50]. A peptidomic study in locusts unveiled the presence of SK in the foregut and hindgut, in addition to the brain, recurrent nerve and esophageal nerves [6]. The distribution of sulfakinin thus appears to be primarily situated in the nervous system, comparable to the sulfakinin receptor transcript distribution observed in *T. castaneum*. Additionally, sulfakinin immunoreactive endocrine cells have been localized in the posterior midgut of the yellow fever mosquito *Aedes aegypti* [50]. Finally, a potential neurohaemal release site for SK has been predicted in *P. americana* [48] which reinforces the possibility that SK might serve as a circulating neurohormone with potential actions in peripheral tissues, such as the fat body or the gut, where receptors have been shown to be present.

### Functional Activity and Dose-response Analysis of *T. castaneum* SK Receptors

**Figure 3:**
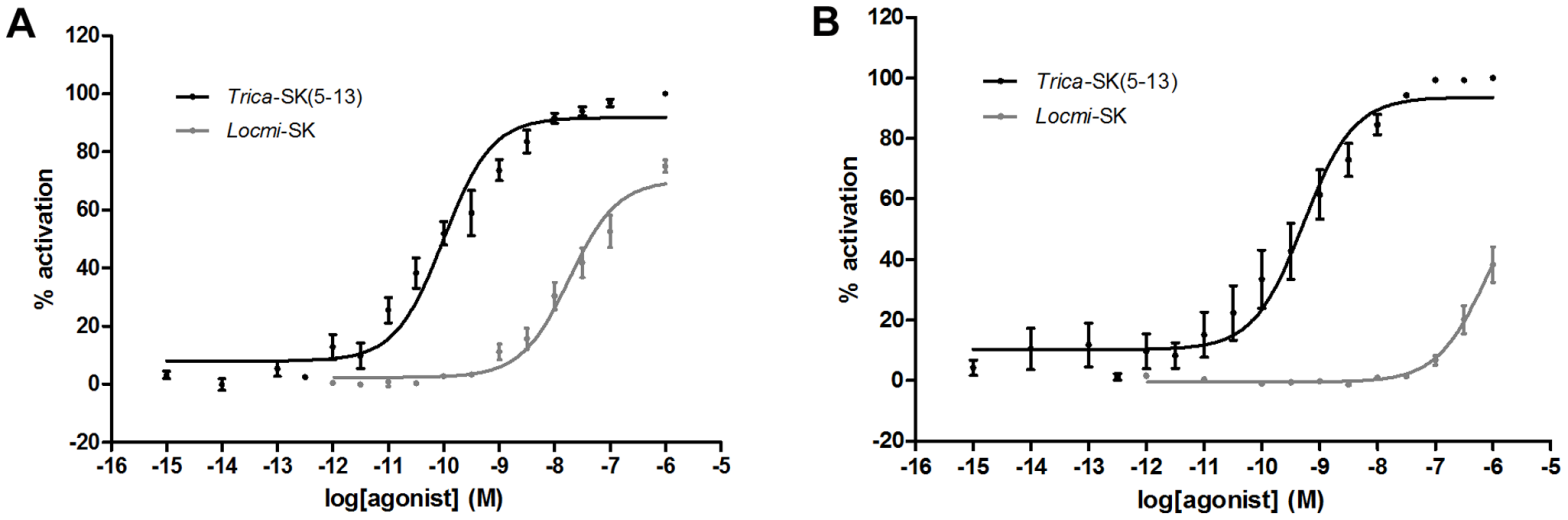
Dose response curves for bioluminescence induced by s*Trica*-SK(5–13) and s*Locmi*-SK in *Trica*-SKR expressing CHO-WTA11 cells. Aequorin bioluminescence induced in CHO-WTA11 cells stably expressing the promiscuous Gα_16_ subunit and transfected with *Trica*-SKR1 (A) or *Trica*-SKR2 (B). Receptor activation shown as the percentage of activation achieved with 1 μM s*Trica*-SK(5–13) (maximal response level = 100%). The zero response level corresponds to treatment of cells with DMEM/BSA. Data represent the mean ± SEM of three independent measurements (each performed in duplicate).

The characterization of both *T. castaneum* SK receptors was first carried out in CHO-WTA11 cells stably expressing apoaequorin and the promiscuous Gα_16_ subunit. A total of 29 different peptides were tested for receptor activation at a concentration of 1 μM. In cells that were transfected with empty pcDNA3.1/V5-His-TOPO TA expression vector construct, no signal was observed upon addition of any of the peptides used in this study. After an initial screen, two peptide agonists were selected for more detailed dose-response analyses on both receptors, namely the sulfated C-terminal fragment of *T. castaneum* SK comprising amino acids 5–13 (s*Trica*-SK(5–13); FDDY(SO_3_)GHMRFamide) and sulfated SK from the migratory locust, *Locusta migratoria* (s*Locmi*-SK; pQLASDDY(SO_3_)GHMRFamide). The dose-response relationships for these two peptides were examined at a concentration range spanning from 1 fM to 1 μM. The resulting sigmoidal curves show a clear dose-dependent and saturable activation signal for both cloned receptors (Figure 3). Calculated EC_50_ values for the *Trica*-SKR1 are 99.5±28.0 pM (95% confidence interval) for s*Trica*-SK(5–13) and 16.8±4.6 nM for s*Locmi*-SK. Half–maximal activation of the *Trica*-SKR2 is elevated in comparison to the *Trica*-SKR1 with EC_50_ values of 524.3±199.7 pM for s*Trica*-SK(5–13) and 775.6±442.7 nM for s*Locmi*-SK. Maximal response for s*Trica*-SK(5–13) was attained at a concentration of 100 nM for both receptors, while s*Locmi*-SK did not reach the maximum response level attained by s*Trica*-SK(5–13) at concentrations up to 1 μM. s*Locmi*-SK was about 150 times less potent than s*Trica*-SK(5–13) in activating the *Trica*-SKR1 and *ca.* 1500 times less potent as *Trica*-SKR2 agonist. The C-terminal active core sequence of both peptides is identical, but the N-terminal part of s*Locmi*-SK probably interferes with optimal *T. castaneum* SK receptor binding. *T. castaneum* is only the second insect species in which dose-response analysis of a SK receptor has been performed. Previously, in *D. melanogaster*, two SK receptors have been deorphanized by different approaches. A β-arrestin translocation assay was used to confirm the activation of the *D. melanogaster* CCKLR-17D1 by sulfated drosulfakinin I and II, but no dose-response analysis was performed during these experiments [20]. Multiple mammalian cell lines, similar to the ones used in this study were implemented to characterize the *D. melanogaster* SK receptor 1. EC_50_ values for receptor activation were in the low nanomolar range in three different cell lines, but were attained using [Leu^7^] drosulfakinin 1 and not the native SK [34]. EC_50_ values in the picomolar range occur occasionally in characterization of insect neuropeptide GPCRs [51]–[54], indicating that these sulfakinin and other insect neuropeptide receptors can be very sensitive for their respective ligands.

### Pharmacological Analysis of *T. castaneum* SK Receptors

Further characterization of the requirements for ligands to activate the receptor was done using a repertoire of 27 additional SK analogs. These peptides were tested for receptor activation at a concentration of 1 μM. A maximum response level was obtained for both SK receptor types when these were stimulated with s*Trica*-SK(5–13) at this concentration (Figure 3). The percentages of activation, relative to this maximum level, attained by these peptides for each receptor are listed in Table 2. The study indicates that the presence or absence of a sulfated tyrosine residue in the ligand is an important parameter that drastically influences SK receptor agonism. Sulfated peptides that do not have any major modifications in the C-terminal core sequence can activate one or both receptors for at least 50% of the response level reached by s*Trica*-SK(5–13) at 1 μM. In contrast, only two nonsulfated peptides were able to attain more than 50% on one receptor. Compound 1070[φ2]wp-2 reached 59% activation on *Trica*-SKR1 and compound 1011[φ2a]wp-7-4 activated *Trica*-SKR2 for 57% when compared to the maximum response level. Compound 1070[φ2]wp-2 contains an aminosuberic acid group that mimics the sulfated tyrosine moiety (Figure S3) [55]. This explains why this peptide was able to activate the receptor quite efficiently. Compound 1011[φ2a]wp-7-4 is the nonsulfated (ns) drosulfakinin 2 (GGDDQFDDYGHMRFamide), which probably resembles the native *Trica*-SK most in its primary structure. Like drosulfakinin 2, native *Trica*-SK contains an N-terminal glycine residue, two acidic residues in the N-terminal part of the peptide and the C-terminal nonapeptide FDDYGHMRFa. When compared to the nonsulfated (ns) *Trica*-SK(5–13), compound 1011[φ2a]wp-7-4 activated both *Trica*-SK receptors to a higher extent. Possibly, this is due to the presence of two aspartic acid residues that might stabilize the binding of the peptide in a similar manner as the glutamic acid residues in the full-length *Trica*-SK.

A partial alanine scan of ns*Trica*-SK revealed several important residues for receptor activation. When any of the C-terminal tetrapeptide residues was replaced by an alanine, the remaining activity of the receptor was almost completely abolished. The HMRFamide C-terminus thus appears to be essential for efficient ligand-mediated activation of both *T. castaneum* SK receptors. Replacement of the glycine residue by an alanine residue did not seem to affect receptor activation, suggesting that the presence of a small, neutral amino acid in the position prior to the HMRFamide suffices for receptor activation. Replacement of the tyrosine residue by an alanine caused a significant drop in the detected bioluminescent response. The importance of the aromatic ring structure in ligand binding and activation of vertebrate CCK receptors has already been evidenced [42], and it is plausible that this aromatic ring is also important for high affinity binding of SKs to their respective receptors, even without the sulfate moiety being present. Receptor activation tests with truncated analogs revealed that the tetrapeptide HMRFamide can activate both receptors to a slightly better extent than nonsulfated FDDYGHMRFamide, while the intermediate truncated analogs in this series seemed to partially or almost entirely lose their potential to activate the *T. castaneum* SK receptors.

Studies on the binding of CCK with its receptors in vertebrates have revealed a lot of direct interaction sites between the neuropeptide ligand and amino acid residues of the receptor. The C-terminal WMDF tetrapeptide of CCK almost entirely fits in the binding pocket that is created by the seven transmembrane helices of the CCK receptor, while more N-terminally located residues interact with the ECLs of the receptor [56]. A possible explanation for the conserved capacity of HMRFamide to activate the receptor in comparison to longer truncated analogs might lie in the fact that this tetrapeptide may fit well in the pocket created by the transmembrane helices, while several of the longer analogs might be sterically hampered and lack additional structural features to allow for a sufficient fit with the ECLs in order to compensate for this. The analysis of a peptide with an additional truncation yielding MRFamide revealed that the histidine group is important in *T. castaneum* SK receptor activation, since almost all activity was lost for both SK receptors.

In this study, a number of sulfated peptides from the nematode *C. elegans* were tested for activation of both *T. castaneum* SK receptors. First and foremost, both sulfated SK-like peptides that are encoded by the neuropeptide-like protein-12 (nlp-12) cDNA were screened (compounds 1678A[φ1]wp-4 and 1678B[φ1]wp-6). These peptides, together with their corresponding receptor, are nematode homologs of vertebrate CCK and insect SK and their receptors, respectively [35]. The C-terminal active core sequence of these peptides is Y(SO_3_)RPLQFamide, and thus only the sulfated tyrosine residue and the C-terminal Famide are identical to insect SK. Nevertheless, these peptides were still capable of activating both *T. castaneum* SK receptors to a similar extent as ns*Trica*-SK and HMRFa. The presence of a sulfated tyrosine and C-terminal Famide group appears to provide some potential to these peptides as agonists for insect SK receptors, although insect Y(SO_3_)GHMRFamides still prove to be a lot more potent. A second set of sulfated neuropeptides with the C-terminal consensus sequences Y(SO_3_)DRPIMAFamide or Y(SO_3_)DRDIMSFamide, derived from *C. elegans* nlp-13, were also assessed for activation of both SK receptors (compounds 1432-2[φ1]wp-6, 1567[φ1]wp-5, 1569[φ2]wp-4 and 1679[φ1]wp-4). The sulfated tyrosine and C-terminal MXFamide of these peptides still provided them with some agonistic potency, but the rest of the C-terminal core differed too much from the insect SKs to reach a response level comparable to YGHMRFamides, although peptide 1567[φ1]wp-5 still approximated the effect of ns*Trica*-SK. The increased number of residues situated between the sulfated tyrosine and the Famide terminus possibly accounts for an extra drop in activation potential of these peptides when compared to the ones encoded by nlp-12.

Three peptides containing the non-naturally occurring amino acid norleucine were tested for receptor activation as well (compounds 1591-1[φ1]wp-3, 1598-2[φ2]wp-4 and 1658[φ1]wp-9). Replacement of methionine with norleucine in the active core of SKs can lead to the retention of biological activity and thus improve stability of SKs [57]. Our study reveals that norleucine is also a good mimic for methionine in the receptor assay. A sulfated peptide containing the C-terminal heptapeptide of *Trica*-SK with a norleucine instead of methionine activated both SK receptors to a similar extent as s*Trica*-SK(5–13). Two other sulfated norleucine containing analogs also elicited over 50% activation of at least one of the SK receptors, despite some changes in their C-terminal core structure. The replacement of arginine with lysine seemed to reduce the potency of these peptides for receptor activation by a small margin. Another peptide containing a non-naturally occurring group in its primary structure was compound 1070[φ2]wp-2. This peptide contains the amino diacid aminosuberic acid to serve as biostable mimic for the sulfated tyrosine [55] and, at 1μM, it activated both receptors approximately to an extent of about 50% of the maximal response level. Aminosuberic acid thus can serve as a substitute for a hydrolysis susceptible sulfated tyrosine group, but may cause a slight drop in potency.

In conclusion, this set of peptides has hinted us about the structural requirements for *T. castaneum* SK receptor agonists. The sulfate moiety is essential for efficient receptor activation. An alanine scan revealed that the most important residues in the amino acid backbone are Y, H, M, R and F. In addition, a set of truncated analogs showed that the tetrapeptide HMRFamide is a stronger agonist of both receptors than many of its N-terminally extended nonsulfated analogs. Furthermore, norleucine can be incorporated as a stable mimic of methionine in the C-terminal HMRFamide tetrapeptide and aminosuberic acid can replace the sulfated tyrosine group, although this causes a drop in receptor activation potential. When compared to ligand properties of vertebrate CCK receptors, a few possible parallels between important amino acid residues in the peptide agonists of SK and CCK receptors were pointed out. Further research on other insect SK receptors with a more diverse array of peptides may shed more light on the interaction and co-evolution between SKs and their respective GPCRs.

### Downstream Signaling Properties of *T. castaneum* SK Receptors

**Figure 4:**
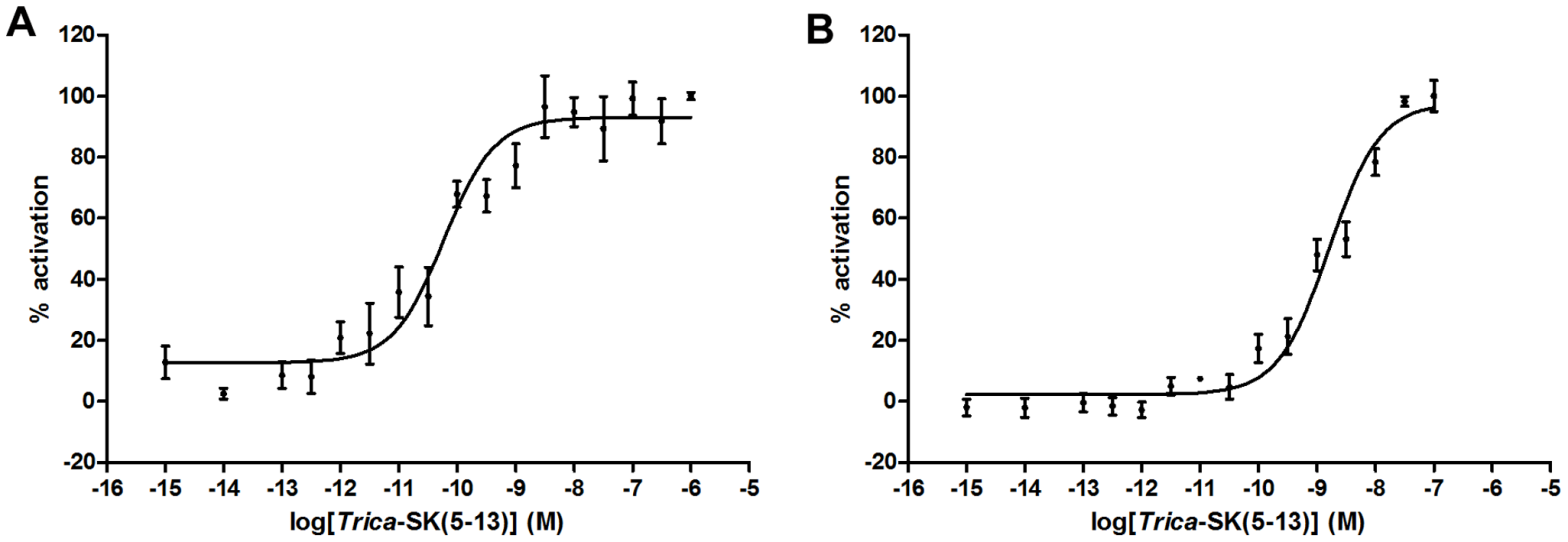
Dose response curves for bioluminescence induced by s*Trica*-SK(5–13) in *Trica*-SKR expressing CHO-PAM28 cells. Aequorin bioluminescence induced in CHO-PAM28 cells transfected with *Trica*-SKR1 (A) or *Trica*-SKR2 (B). Receptor activation shown as the percentage of activation achieved with 1 μM *sTrica*-SK(5–13) (maximal response level = 100%). The zero response level corresponds to treatment of cells with DMEM/BSA. Data represent the mean ± SEM of three independent measurements (each performed in duplicate).

The intracellular signaling properties of both *T. castaneum* SK receptors were characterized using CHO-PAM28 cells and HEK293T cells and s*Trica*-SK(5–13) as ligand. In both CHO-PAM28 and HEK293T cells that were transfected with empty pcDNA3.1/V5-His-TOPO TA expression vector construct, no signal was observed upon addition to s*Trica*-SK(5–13). CHO-PAM28 cells stably express apoaequorin, but do not express the promiscuous Gα_16_ protein. Both *T. castaneum* SK receptors caused a dose-dependent increase in aequorin bioluminescence upon binding of s*Trica*-SK(5–13), indicating that the endogenous Gα_q_ in these cells can couple these receptors to a Ca^2+^ response (Figure 4). EC_50_ values for s*Trica*-SK(5–13) induced receptor activation in CHO-PAM28 cells are 58.52±23.97 pM for *Trica*-SKR1 and 1.61±0.45 nM for *Trica*-SKR2. To our knowledge this is the first time that an insect SK receptor has been shown to activate the Ca^2+^ pathway. We must, however, remain careful in extrapolating the results from cell-based receptor studies. Results obtained in cell lines may not accurately reflect all situations that occur *in vivo*, but provide us with a hint regarding the signaling properties of these insect SK receptor proteins.

**Figure 5:**
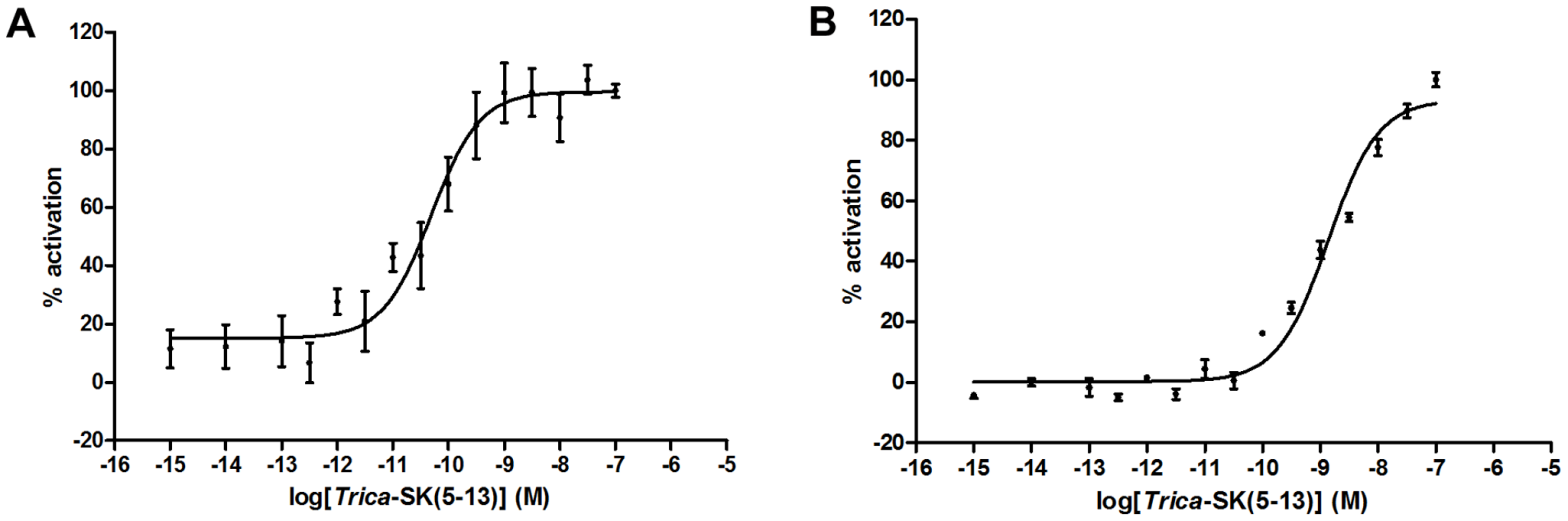
Dose response curves for bioluminescence induced by s*Trica*-SK(5–13) in *Trica*-SKR expressing HEK293T cells. Luciferase bioluminescence induced in HEK293T cells transfected with *Trica*-SKR1 (A) or *Trica*-SKR2 (B) and CRE-luciferase construct. Receptor activation shown as the percentage of activation achieved with 100 nM *sTrica*-SK(5–13) (maximal response level = 100%). The zero response level corresponds to treatment of cells with DMEM/IBMX. Data represent the mean ± SEM of six (SKR1) or three (SKR2) independent measurements (each performed in duplicate).

In addition to testing in CHO-PAM28 cell lines, the signaling characteristics of *T. castaneum* SK receptors were also examined in HEK293T cells to assess their possible effects on cellular levels of the second messenger, cAMP. HEK293T cells were cotransfected with *T. castaneum* SK receptor and a CRE-luciferase reporter construct to detect changes in cAMP levels. In this cellular assay system, both SK receptors showed an increase of luciferase bioluminescence upon binding of s*Trica*-SK(5–13) (Figure 5). Both *T. castaneum* SK receptors thus appear to couple positively to cAMP in this *in vitro* cell system and probably use the Gα_s_ subunit of the associated G-protein to achieve this. Calculated EC_50_ values were 4.9±4.2 pM for the *Trica*-SKR1 and 1.4±0.3 nM for the *Trica*-SKR2. Positive coupling of an insect SK receptor to cAMP was previously demonstrated in *D. melanogaster* [33]. Although the results from our *in vitro* characterization of second messenger pathways may depend on the cell line used and do not necessarily reflect all *in vivo* situations, this receptor analysis in HEK293T cells is in line with earlier findings on SK induced effects.

This study indicates that both *T. castaneum* SK receptors appear to display dual coupling characteristics towards the cAMP and Ca^2+^ second messenger systems when stimulated with SK agonist. The signaling properties of these SK receptors seem to resemble these of vertebrate type 1 CCK receptors that also can stimulate both Ca^2+^ and cAMP through Gα_q_ and Gα_s_ respectively. This is in contrast to type 2 CCK receptors that do not transduce their signal via Gα_s_ [58]. Our results further affirm the functional and structural homology between SK and CCK receptors to the level of peptide agonist requirements for receptor activation and second messenger pathways. This detailed pharmacological information can be beneficial for future *in vitro* and *in vivo* studies concerning SK signaling pathways and their possible applications in pest management.

## Supporting Information

Figure S1 **Amino acid sequence of the** ***Trica*** **-SKR1.** The additional glutamate residue in comparison to the GenBank sequence is tagged with a dot. The cysteine residues that can form the stabilizing disulfide bridge are marked with a vertical dotted line. Residues marked in grey indicate putative intracellular phosphorylation sites; A indicates possible phosphorylation by PKA, C designates a putative PKC phosphorylation site and G indicates possible phosphorylation by GRKs. Putative N-linked glycosylation sites on the ECLs are indicated by a reverse triangle, while putative palmitoylation sites are marked by stars. The Met and Arg residue that are important for interaction with the sulfated tyrosine residue are marked with a square.
(TIF)

Figure S2 **Amino acid sequence of the** ***Trica*** **-SKR2.** The cysteine residues that can form the stabilizing disulfide bridge are marked with a vertical dotted line. Residues marked in grey indicate putative intracellular phosphorylation sites; A indicates possible phosphorylation by PKA, C designates a putative PKC phosphorylation site and G indicates possible phosphorylation by GRKs. Putative N-linked glycosylation sites on the ECLs are indicated by a reverse triangle. The Arg residue that is important for interaction with the sulfate group is marked with a square.
(TIF)

Figure S3 **Comparison of molecular structures of sulfated tyrosine and aminosuberic acid.**
(TIF)

## References

[1] Spit J, Badisco L, Verlinden H, Van Wielendaele P, Zels S, et al (2012) Peptidergic control of food intake and digestion in insects. Canadian Journal of Zoology-Revue Canadienne de Zoologie 90: 489–506.

[2] Verleyen P, Huybrechts J, Sas F, Clynen E, Baggerman G, et al (2004) Neuropeptidomics of the grey flesh fly, *Neobellieria bullata* . Biochemical and Biophysical Research Communications 316: 763–770. 15033466 10.1016/j.bbrc.2004.02.115

[3] Audsley N, Matthews HJ, Down RE, Weaver RJ (2011) Neuropeptides associated with the central nervous system of the cabbage root fly, *Delia radicum* (L). Peptides 32: 434–440. 20869420 10.1016/j.peptides.2010.08.028

[4] Zoephel J, Reiher W, Rexer KH, Kahnt J, Wegener C (2012) Peptidomics of the Agriculturally Damaging Larval Stage of the Cabbage Root Fly *Delia radicum* (Diptera: Anthomyiidae). Plos One 7: e41543. 22848525 10.1371/journal.pone.0041543 PMC3405134

[5] Li B, Predel R, Neupert S, Hauser F, Tanaka Y, et al (2008) Genomics, transcriptomics, and peptidomics of neuropeptides and protein hormones in the red flour beetle *Tribolium castaneum* . Genome Research 18: 113–122. 18025266 10.1101/gr.6714008 PMC2134770

[6] Clynen E, Schoofs L (2009) Peptidomic survey of the locust neuroendocrine system. Insect Biochemistry and Molecular Biology 39: 491–507. 19524670 10.1016/j.ibmb.2009.06.001

[7] Hauser F, Neupert S, Williamson M, Predel R, Tanaka Y, et al (2010) Genomics and Peptidomics of Neuropeptides and Protein Hormones Present in the Parasitic Wasp *Nasonia vitripennis* . Journal of Proteome Research 9: 5296–5310. 20695486 10.1021/pr100570j

[8] Huybrechts J, Bonhomme J, Minoli S, Prunier-Leterme N, Dombrovsky A, et al (2010) Neuropeptide and neurohormone precursors in the pea aphid, *Acyrthosiphon pisum* . Insect Molecular Biology 19: 87–95. 10.1111/j.1365-2583.2009.00951.x 20482642

[9] Ons S, Richter F, Urlaub H, Pomar RR (2009) The neuropeptidome of *Rhodnius prolixus* brain. Proteomics 9: 788–792. 19137558 10.1002/pmic.200800499

[10] Schoofs L, Janssen T, Nachman RJ (2013) Sulfakinins. In: Handbook of Biologically Active Peptides. 310–314.

[11] Predel R, Brandt W, Kellner R, Rapus J, Nachman RJ, et al (1999) Post-translational modifications of the insect sulfakinins: sulfation, pyroglutamate-formation and O-methylation of glutamic acid. Eur J Biochem 263: 552–560. 10406966 10.1046/j.1432-1327.1999.00532.x

[12] Nachman RJ, Holman GM, Cook BJ, Haddon WF, Ling N (1986) Leucosulfakinin-II, a blocked sulfated insect neuropeptide with homology to cholecystokinin and gastrin. Biochem Biophys Res Commun 140: 357–364. 3778455 10.1016/0006-291x(86)91098-3

[13] Nachman RJ, Holman GM, Haddon WF, Ling N (1986) Leucosulfakinin, a sulfated insect neuropeptide with homology to gastrin and cholecystokinin. Science 234: 71–73. 3749893 10.1126/science.3749893

[14] Nichols R, Schneuwly SA, Dixon JE (1988) Identification and characterization of a *Drosophila* homologue to the vertebrate neuropeptide cholecystokinin. J Biol Chem 263: 12167–12170. 2842322

[15] Veenstra JA (1989) Isolation and structure of two gastrin/CCK-like neuropeptides from the American cockroach homologous to the leucosulfakinins. Neuropeptides 14: 145–149. 2615921 10.1016/0143-4179(89)90038-3

[16] Fonagy A, Schoofs L, Proost P, Van Damme J, De Loof A (1992) Isolation and primary structure of two sulfakinin-like peptides from the fleshfly, *Neobellieria bullata* . Comp Biochem Physiol C 103: 135–142. 1360367 10.1016/0742-8413(92)90242-y

[17] Maestro JL, Aguilar R, Pascual N, Valero ML, Piulachs MD, et al (2001) Screening of antifeedant activity in brain extracts led to the identification of sulfakinin as a satiety promoter in the German cockroach. Are arthropod sulfakinins homologous to vertebrate gastrins-cholecystokinins? Eur J Biochem 268: 5824–5830. 11722569 10.1046/j.0014-2956.2001.02527.x

[18] Palmer GC, Tran T, Duttlinger A, Nichols R (2007) The drosulfakinin 0 (DSK 0) peptide encoded in the conserved Dsk gene affects adult *Drosophila melanogaster* crop contractions. J Insect Physiol 53: 1125–1133. 17632121 10.1016/j.jinsphys.2007.06.001

[19] Nichols R, Manoogian B, Walling E, Mispelon M (2009) Plasticity in the effects of sulfated and nonsulfated sulfakinin on heart contractions. Front Biosci 14: 4035–4043. 10.2741/3510 19273332

[20] Chen X, Peterson J, Nachman RJ, Ganetzky B (2012) Drosulfakinin activates CCKLR-17D1 and promotes larval locomotion and escape response in *Drosophila* . Fly (Austin ) 6: 290–297. 22885328 10.4161/fly.21534 PMC3519664

[21] Nichols R (2007) The first nonsulfated sulfakinin activity reported suggests nsDSK acts in gut biology. Peptides 28: 767–773. 17292511 10.1016/j.peptides.2007.01.009

[22] Marciniak P, Kuczer M, Rosinski G (2011) New physiological activities of myosuppressin, sulfakinin and NVP-like peptide in *Zophobas atratus* beetle. J Comp Physiol B 181: 721–730. 21409564 10.1007/s00360-011-0563-5 PMC3140940

[23] Wei Z, Baggerman G, Nachman RJ, Goldsworthy G, Verhaert P, et al (2000) Sulfakinins reduce food intake in the desert locust, *Schistocerca gregaria* . J Insect Physiol 46: 1259–1265. 10844144 10.1016/s0022-1910(00)00046-9

[24] Downer KE, Haselton AT, Nachman RJ, Stoffolano JG Jr (2007) Insect satiety: sulfakinin localization and the effect of drosulfakinin on protein and carbohydrate ingestion in the blow fly, *Phormia regina* (Diptera: Calliphoridae). J Insect Physiol 53: 106–112. 17166511 10.1016/j.jinsphys.2006.10.013

[25] Yu N, Benzi V, Zotti MJ, Staljanssens D, Kaczmarek K, et al (2013) Analogs of sulfakinin-related peptides demonstrate reduction in food intake in the red flour beetle, *Tribolium castaneum*, while putative antagonists increase consumption. Peptides 41: 107–112. 23246802 10.1016/j.peptides.2012.12.005

[26] Meyering-Vos M, Muller A (2007) RNA interference suggests sulfakinins as satiety effectors in the cricket *Gryllus bimaculatus* . J Insect Physiol 53: 840–848. 17560597 10.1016/j.jinsphys.2007.04.003

[27] Yu N, Smagghe G (2013) Characterization of sulfakinin receptor 2 and its role in food intake in the red flour beetle, *Tribolium castaneum*. Peptides. S0196–9781 (13). 10.1016/j.peptides.2013.12.011 24373934

[28] Yu N, Nachman RJ, Smagghe G (2013) Characterization of sulfakinin and sulfakinin receptor and their roles in food intake in the red flour beetle *Tribolium castaneum* . Gen Comp Endocrinol 188: 196–203. 23524001 10.1016/j.ygcen.2013.03.006

[29] Nachman RJ, Giard W, Favrel P, Suresh T, Sreekumar S, et al (1997) Insect myosuppressins and sulfakinins stimulate release of the digestive enzyme α-amylase in two invertebrates: the scallop *Pecten maximus* and insect *Rynchophorus ferrugineus* . Ann N Y Acad Sci 814: 335–338.

[30] Harshini S, Nachman RJ, Sreekumar S (2002) *In vitro* release of digestive enzymes by FMRF amide related neuropeptides and analogues in the lepidopteran insect *Opisina arenosella* (Walk.). Peptides 23: 1759–1763. 12383863 10.1016/s0196-9781(02)00152-3

[31] Nichols R, Egle JP, Langan NR, Palmer GC (2008) The different effects of structurally related sulfakinins on *Drosophila melanogaster* odor preference and locomotion suggest involvement of distinct mechanisms. Peptides 29: 2128–2135. 18786583 10.1016/j.peptides.2008.08.010 PMC3430133

[32] Kiss B, Szlanka T, Zvara A, Zurovec M, Sery M, et al (2013) Selective elimination/RNAi silencing of FMRF-related peptides and their receptors decreases the locomotor activity in *Drosophila melanogaster* . Gen Comp Endocrinol 191: 137–145. 23770020 10.1016/j.ygcen.2013.05.023

[33] Chen X, Ganetzky B (2012) A neuropeptide signaling pathway regulates synaptic growth in *Drosophila* . J Cell Biol 196: 529–543. 22331845 10.1083/jcb.201109044 PMC3283997

[34] Kubiak TM, Larsen MJ, Burton KJ, Bannow CA, Martin RA, et al (2002) Cloning and functional expression of the first *Drosophila melanogaster* sulfakinin receptor DSK-R1. Biochem Biophys Res Commun 291: 313–320. 11846406 10.1006/bbrc.2002.6459

[35] Janssen T, Meelkop E, Lindemans M, Verstraelen K, Husson SJ, et al (2008) Discovery of a cholecystokinin-gastrin-like signaling system in nematodes. Endocrinology 149: 2826–2839. 18339709 10.1210/en.2007-1772

[36] Beeman RW, Haas S, Friesen K (2009) Beetle wrangling tips. (An introduction to the care and handling of *Tribolium castaneum*). URL: http://bru.gmprc.ksu.edu/proj/tribolium/wrangle.asp (Last modified on 21.08.2012).

[37] Vandesompele J, De Preter K, Pattyn F, Poppe B, Van Roy N, et al (2002) Accurate normalization of real-time quantitative RT-PCR data by geometric averaging of multiple internal control genes. Genome Biol 3: RESEARCH0034. 12184808 10.1186/gb-2002-3-7-research0034 PMC126239

[38] Vleugels R, Lenaerts C, Baumann A, Vanden Broeck J, Verlinden H (2013) Pharmacological characterization of a 5-HT1-type serotonin receptor in the red flour beetle, *Tribolium castaneum* . PLoS One 8: e65052. 23741451 10.1371/journal.pone.0065052 PMC3669024

[39] Nachman RJ, Isaac RE, Coast GM, Holman GM (1997) Aib-containing analogues of the insect kinin neuropeptide family demonstrate resistance to an insect angiotensin-converting enzyme and potent diuretic activity. Peptides 18: 53–57. 9114452 10.1016/s0196-9781(96)00233-1

[40] Miller LJ, Gao F (2008) Structural basis of cholecystokinin receptor binding and regulation. Pharmacol Ther 119: 83–95. 18558433 10.1016/j.pharmthera.2008.05.001 PMC2570585

[41] Gigoux V, Maigret B, Escrieut C, Silvente-Poirot S, Bouisson M, et al (1999) Arginine 197 of the cholecystokinin-A receptor binding site interacts with the sulfate of the peptide agonist cholecystokinin. Protein Sci 8: 2347–2354. 10595537 10.1110/ps.8.11.2347 PMC2144185

[42] Gigoux V, Escrieut C, Silvente-Poirot S, Maigret B, Gouilleux L, et al (1998) Met-195 of the cholecystokinin-A receptor interacts with the sulfated tyrosine of cholecystokinin and is crucial for receptor transition to high affinity state. J Biol Chem 273: 14380–14386. 9603948 10.1074/jbc.273.23.14380

[43] Wicher D, Derst C, Gautier H, Lapied B, Heinemann SH, et al (2007) The satiety signaling neuropeptide perisulfakinin inhibits the activity of central neurons promoting general activity. Front Cell Neurosci 1: 3. 18946521 10.3389/neuro.03.003.2007 PMC2525928

[44] Roeder T (2005) Tyramine and octopamine: ruling behavior and metabolism. Annu Rev Entomol 50: 447–477. 15355245 10.1146/annurev.ento.50.071803.130404

[45] Arrese EL, Soulages JL (2010) Insect fat body: energy, metabolism, and regulation. Annu Rev Entomol 55: 207–225. 19725772 10.1146/annurev-ento-112408-085356 PMC3075550

[46] Duve H, Thorpe A, Scott AG, Johnsen AH, Rehfeld JF, et al (1995) The sulfakinins of the blowfly *Calliphora vomitoria*. Peptide isolation, gene cloning and expression studies. Eur J Biochem 232: 633–640. 7556217 10.1111/j.1432-1033.1995.tb20854.x

[47] Nichols R, McCormick J, Lim I (1997) Dromyosuppressin and drosulfakinin, two structurally related *Drosophila* neuropeptides, are uniquely expressed in the adult central nervous system. Ann N Y Acad Sci 814: 315–318. 9160985 10.1111/j.1749-6632.1997.tb46173.x

[48] East PD, Hales DF, Cooper PD (1997) Distribution of sulfakinin-like peptides in the central and sympathetic nervous system of the American cockroach, *Periplaneta americana* (L.) and the field cricket, *Teleogryllus commodus* (Walker). Tissue Cell 29: 347–354. 9225486 10.1016/s0040-8166(97)80010-9

[49] Söderberg JA, Carlsson MA, Nässel DR (2012) Insulin-Producing Cells in the *Drosophila* Brain also Express Satiety-Inducing Cholecystokinin-Like Peptide, Drosulfakinin. Front Endocrinol (Lausanne) 3: 109. 22969751 10.3389/fendo.2012.00109 PMC3431609

[50] Veenstra JA, Lau GW, Agricola HJ, Petzel DH (1995) Immunohistological Localization of Regulatory Peptides in the Midgut of the Female Mosquito *Aedes aegypti* . Histochemistry and Cell Biology 104: 337–347. 8574883 10.1007/BF01458127

[51] Horodyski FM, Verlinden H, Filkin N, Vandersmissen HP, Fleury C, et al (2011) Isolation and functional characterization of an allatotropin receptor from *Manduca sexta* . Insect Biochem Mol Biol 41: 804–814. 21699978 10.1016/j.ibmb.2011.06.002

[52] Verlinden H, Lismont E, Bil M, Urlacher E, Mercer A, et al (2013) Characterisation of a functional allatotropin receptor in the bumblebee, *Bombus terrestris* (Hymenoptera, Apidae). Gen Comp Endocrinol 193: 193–200. 23968772 10.1016/j.ygcen.2013.08.006

[53] Vuerinckx K, Verlinden H, Lindemans M, Vanden Broeck J, Huybrechts R (2011) Characterization of an allatotropin-like peptide receptor in the red flour beetle, *Tribolium castaneum* . Insect Biochem Mol Biol 41: 815–822. 21742031 10.1016/j.ibmb.2011.06.003

[54] Shi Y, Huang H, Deng X, He X, Yang J, et al (2011) Identification and functional characterization of two orphan G-protein-coupled receptors for adipokinetic hormones from silkworm *Bombyx mori* . J Biol Chem 286: 42390–42402. 22009754 10.1074/jbc.M111.275602 PMC3234951

[55] Nachman RJ, Vercammen T, Williams H, Kaczmarek K, Zabrocki J, et al (2005) Aliphatic amino diacid Asu functions as an effective mimic of Tyr(SO_3_H) in sulfakinins for myotropic and food intake-inhibition activity in insects. Peptides 26: 115–120. 15626511 10.1016/j.peptides.2004.07.018

[56] Fourmy D, Escrieut C, Archer E, Gales C, Gigoux V, et al (2002) Structure of cholecystokinin receptor binding sites and mechanism of activation/inactivation by agonists/antagonists. Pharmacol Toxicol 91: 313–320. 12688374 10.1034/j.1600-0773.2002.910608.x

[57] Nachman RJ, Holman GM, Haddon WF (1988) Structural aspects of gastrin/CCK-like insect leucosulfakinins and FMRF-amide. Peptides 9 Suppl 1 137–143. 2908810 10.1016/0196-9781(88)90237-9

[58] Dufresne M, Seva C, Fourmy D (2006) Cholecystokinin and gastrin receptors. Physiol Rev 86: 805–847. 16816139 10.1152/physrev.00014.2005

