# Supplementary figures and images for: Signaling Properties and Pharmacological Analysis of Two Sulfakinin Receptors from the Red Flour Beetle, Tribolium castaneum
Source: PLoS One. 2014 Apr 9;9(4):e94502. doi: 10.1371/journal.pone.0094502 (PMC3981819; doi:10.1371/journal.pone.0094502)

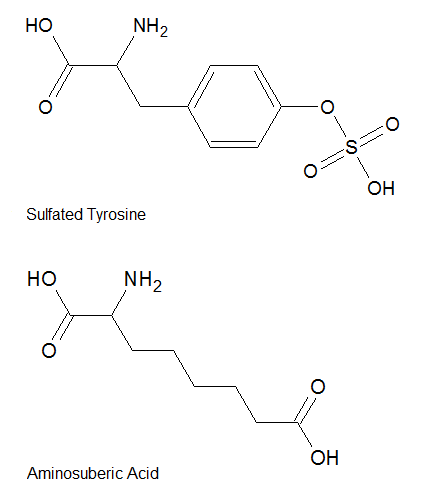

Supplement: Figure S3 — Comparison of molecular structures of sulfated tyrosine and aminosuberic acid. (TIF) [file pone.0094502.s003.tif]
